# Long-Term Prognostic Significance of High-Sensitive Troponin I Increase during Hospital Stay in Patients with Acute Myocardial Infarction and Non-Obstructive Coronary Arteries

**DOI:** 10.3390/medicina56090432

**Published:** 2020-08-27

**Authors:** Magdalena Jędrychowska, Rafał Januszek, Wojciech Wańha, Krzysztof Piotr Malinowski, Piotr Kunik, Agata Trznadel, Joanna Bartuś, Bartłomiej Staszczak, Sławomir Mateusz Januszek, Tomasz Kameczura, Wojciech Wojakowski, Andrzej Surdacki, Stanisław Bartuś

**Affiliations:** 1 Department of Cardiology and Cardiovascular Interventions, University Hospital, 30-688 Kraków, Poland; magdalena.mj.mj@gmail.com; 2 Department of Clinical Rehabilitation, University of Physical Education, 31-571 Kraków, Poland; 3 Department of Cardiology and Structural Heart Diseases, Medical University of Silesia, 40-055 Katowice, Poland; wojciech.wanha@gmail.com (W.W.); piotrkunik@gmail.com (P.K.); trznadelagata@gmail.com (A.T.); wwojakowski@sum.edu.pl (W.W.); 4 Institute of Public Health, Faculty of Health Sciences, Jagiellonian University Medical College, 31-008 Kraków, Poland; krzysztof.piotr.malinowski@gmail.com; 5 Andrzej Frycz Modrzewski Kraków University, 30-705 Kraków, Poland; jbartus@babinski.pl; 6 Jagiellonian University Medical College, 31-008 Kraków, Poland; bartlomiej.staszczak@gmail.com; 7 Gynaecology and Obstetrics Clinic, Clinical District Hospital, 35-310 Rzeszow, Poland; versus_25@tlen.pl; 8 Chair of Electroradiology, Faculty of Medicine, University of Rzeszow, 35-310 Rzeszow, Poland; tomasz_kameczura@yahoo.com; 9 Department of Cardiology, Jagiellonian University Medical College, 40-055 Kraków, Poland; andrzej.surdacki@uj.edu.pl (A.S.); stanislaw.bartus@uj.edu.pl (S.B.); 10 Department of Cardiology and Cardiovascular Interventions, University Hospital, 40-055 Kraków, Poland

**Keywords:** clinical outcomes, myocardial infarction with non-obstructive coronary artery disease, predictors, troponin I

## Abstract

*Background and Objectives:* A topic already widely investigated is the negative prognostic value regarding the extent of high sensitive troponin I (hs-TnI) increases among patients with myocardial infarction (MI) and obstructive coronary atherosclerosis compared to a group of patients with MI and non-obstructive coronary atherosclerosis (MINOCA). Thus, the aim of this study was to evaluate the prognostic value concerning the extent of hs-TnI increase on clinical outcomes among patients with a MINOCA working diagnosis. *Materials and Methods:* We selected 337 consecutive patients admitted to hospital with a working diagnosis of MINOCA. The patients were divided in three groups according to the extent of hs-TnI increase during hospitalization (increase ≤5-times above the limit of the upper norm, >5 and ≤20-times, and >20-times). The study endpoints included all-cause mortality and major adverse cardiac and cerebrovascular events (MACCE; cerebral stroke and transient ischemic attacks, MI, coronary artery revascularization, either percutaneous coronary intervention or coronary artery bypass grafting and all-cause mortality). *Results:* During the mean follow-up period of 516.1 ± 239.8 days, using Kaplan–Meier survival curve analysis, significantly higher mortality rates were demonstrated among patients from the group with the greatest hs-TnI increase compared to the remaining groups (*p* = 0.01) and borderline values for MACCE (*p* = 0.053). Multivariable cox regression analysis did not confirm hs-TnI among factors related to increased MACCE or all-cause mortality rates. *Conclusion:* While a relationship between clinical outcomes and the extent of the hs-TnI increase among patients with a MINOCA working diagnosis remains, it does not seem to be not as strong as it is in patients with obstructive coronary atherosclerosis.

## 1. Introduction

Acute myocardial infarction (AMI) with non-obstructive coronary arteries defined as lack of relevant (over 50%) stenosis in coronary angiography has currently become an interesting topic [1]. The prevalence of myocardial infarction with non-obstructive coronary arteries (MINOCA) among patients admitted to hospital with acute coronary syndrome (ACS) is estimated to be at 6% [2,3]. Diagnosis of MINOCA is considered working, due to its various etiologies often requiring prolonged diagnostics and specific treatment [4]. During five years of observation, the mortality rate may even reach 10.9% in the group of patients with MINOCA [5]. In the ACUITY (“Acute Catheterization and Urgent Intervention Triage Strategy”) trial patients with MINOCA, the 1-year death rate was higher than in the case of non-ST-segment elevation myocardial infarction (NSTEMI) patients with myocardial infarction and obstructive coronary artery disease (MI-CAD), mostly caused by non-cardiac mortality [6]. Various etiologies and complex pathomechanisms of MINOCA make identification of the underlying cause and selection of the proper clinical assessment as well as treatment extremely challenging. The negative predictive value of cardiac troponin in AMI is well-known, however, the impact of high-sensitivity troponin levels on MINOCA patients requires further investigation [7]. Therefore, the current study aimed to characterize patients with a working diagnosis of AMI. These patients demonstrated non-obstructive coronary arteries due to the extent of the high-sensitive troponin I (hs-TnI) increase. Furthermore, we aimed to assess clinical outcomes in terms of all-cause mortality and major adverse cardiac and cerebrovascular events (MACCE).

## 2. Materials and Methods

### 2.1. Study Population

We performed an observational cohort study involving patients hospitalized at two Polish academic cardiology centers. These patients had AMI with non-obstructive coronary arteries confirmed in angiography. The patients were included partially retrospectively and partially on an ongoing basis in a prospective manner. Patients and follow-up data were collected between January 2014 and December 2018. The diagnosis of AMI was made in accordance with the fourth universal definition of myocardial infarction and the 2017 working group position paper on myocardial infarction with non-obstructive coronary arteries by the European Society of Cardiology [8,9]. Also, type 2 myocardial infarction and its individual etiologies have been defined in accordance with the current European guidelines and recommended consensus [8,9]. In the current study, we included all consecutive patients with ACSs at baseline who were qualified for urgent coronary artery angiography. Its result confirmed non-obstructive coronary artery disease, and patients with unstable angina at baseline, based on subsequent determinations of myocardial necrosis markers, were reclassified to the AMI group. The exclusion criteria were qualification for coronary revascularization or the lack of confirmed AMI among patients initially diagnosed with unstable angina. Information on demographic features, cardiovascular risk factors, comorbidities, and medication were collected at admission based on detailed patient interview and medical documentation. Non-stenotic plaque was defined as stenosis in coronary artery angiography less than 50%, which was confirmed by at least two views. The plaque was defined as eccentric when the atherosclerotic plaque failed to involve the entire coronary artery circumference and left variable arc of the disease-free wall. Autoimmune and oncological diseases were diagnosed in the case of previous disease history. Previous or ongoing surgical, pharmacological, or radiation oncological treatment in the case of oncological diseases were diagnosed. Furthermore, confirmed diagnosis of the disease from autoimmunization or previous or toxic immunosuppressive treatment associated with these diseases were also taken into consideration. Takotsubo cardiomyopathy was diagnosed according to the generally accepted recommendations [10]. Coronary artery spasm was defined as a reduction by >50% in luminal diameter assessed by coronary angiography with accompanying symptoms and/or ischemic ST-segment changes compared with post-intracoronary nitroglycerin. The study protocol was approved by the local Bioethics Committee and complied with the declaration of Helsinki. Ordinary written informed consent for coronary angiography and data collection was obtained from the patients. 

### 2.2. Markers of Myocardial Injury

hs-TnI was determined using The ADVIA Centaur^®^ TNIH assay (Siemens Healthcare GmbH, Erlagen, Germany), which is a device using dual-capture sandwich immunoassay using magnetic latex particles and a proprietary acridinium ester (tri-sulfo propyl acridinium ester) for chemiluminescence detection. Due to the fact that the troponin levels were estimated using different assays and upper limits, we calculated the troponin index based on the maximal troponin value measured during hospitalization in order to unify the results. The troponin index was calculated as the ratio of the maximal troponin concentration and the upper limit of the reference range for the particular assay which was based on calculating the 95% upper confidence level. We enrolled 337 consecutive patients and divided them into three groups according to troponin index. The first group included patients with mild (≤5-times above the upper normal limit), the second group with moderate (>5 to ≤20-times above the upper normal limit), and third group with a large hs-TnI increase (>20-times above the upper normal limit).

### 2.3. Working Diagnosis, Etiology, and Discharge Diagnosis

Working diagnosis of particular types of ACSs (AMI or unstable angina) is the concept used in the present work for patients qualified for coronary angiography (from admission to the department until the beginning of coronary angiography). Working diagnosis of MINOCA was set immediately after coronary angiography, when no significant stenosis was found in the coronary arteries, no further revascularization was required, and the diagnosis of AMI was confirmed according to the 4th universal definition of myocardial infarction. During further hospitalization, some patients changed the diagnosis depending on the clinical picture or the results of imaging and biochemical blood tests. Working etiology was set until the end of hospitalization, and it was not recognized as final or most probable etiology, due to the fact that some tests, e.g., cardiac magnetic resonance, blood tests (thrombophilia test package) or neoplastic tests (positron emission tomography) were performed after discharge from hospital and for various reasons were not considered in the final etiology. Discharge diagnosis was the diagnosis made on the discharge card, which was based on the entire clinical picture and research results collected until the patient was discharged from the hospital.

### 2.4. Echocardiography

All study participants underwent comprehensive two-dimensional echocardiography using Doppler and tissue Doppler imaging via commercially available ultrasound systems equipped with harmonic imaging (Vivid 9 or Vivid 95, GEHealthcare, General Electric Corp., Waukesha, WI, USA). The test was performed with the patient in left lateral decubitus position. The left ventricle ejection fraction was calculated based on the fraction area change in two dimensions. The function of the heart valves, the presence of fluid in the pericardial sac, the size of the heart cavities, and the presence of regional contractility disorders were also assessed.

### 2.5. Coronary Angiography

Selective coronary angiography was performed using a standard procedure via radial access. Intracoronary nitrate (100 or 200 mg) was administered before the angiographic views. Quantitative coronary angiography was performed under optimal projections with a computer-assisted coronary angiographic analysis system (Siemens Artis Q with PURE, Siemens Healthcare GmbH, Erlagen, Germany). The coronary arteries were visualized in the left and right oblique planes with cranial and caudal angulations. The operators were not investigators. Qualification for the study was based on the description of the procedures (coronary angiography). Lesions considered angiographically non-qualifying for intravascular treatment, or in the case of borderline lesions, significance was ruled out by additional tests such as fractional coronary flow reserve measurement or intravascular ultrasound. Parietal lesions or smooth contours of the vessels were also considered eligible for the study.

### 2.6. Study Endpoints

Study endpoints were all-cause mortality and MACCE rates, where MACCE included all-cause mortality, cerebral stroke and transient ischemic attacks (TIA), myocardial infarction, coronary artery revascularization, either percutaneous coronary intervention (PCI) or coronary artery bypass grafting (CABG). The all-cause, in-hospital mortality was assessed until discharge from hospital, while the overall all-cause mortality was calculated for the whole period (from admission to hospital until the end of follow-up period). The follow-up was carried out on the basis of documentation from the outpatient clinic, telephone calls and the database of the National Health Fund.

### 2.7. Statistical Analysis

Continuous variables in three selected groups of patients according to the extent of troponin increase were compared applying ANOVA, the Welch or Kruskal–Wallis test, when appropriate. Categorical variables were compared using Pearson’s or Fisher’s test, where applicable. Differences in all-cause mortality rates and the occurrence of MACCE between the three selected groups of patients according to the extent of troponin increase were assessed during the follow-up period via the Kaplan–Meier method and were compared using the log-rank test. All available variables were screened as potential predictors of all-cause mortality as well as MACCE. Multivariable models were constructed from all variables which were considered significant according to univariable analysis using stepwise regression with minimization of Bayes information criterion (BIC) as a target. BIC was chosen because it penalizes the complexity of models expressed as a number of parameters. Therefore, it favors less complex models with a smaller number of predictors and thus, provides some protection against over-parameterization. In a case of correlation of predictors, the stronger was favored for inclusion in the model. Multi collinearity was assessed using variance inflation factors (VIF). Assumptions of cox regression, i.e., proportional hazards were tested. The C-statistic was used as a measure of goodness-of-fit. Final models were validated using bootstraping with 1000 repetitions. Bootstrapped bias-corrected C-statistics were also presented. Results from all univariable as well as multivariable models were presented as risk ratios with 95% confidence intervals (CI). All of the analyses were performed using the SAS 9.1 system (SAS Institute Inc., Cary, NC, USA). All statistical tests were two-sided (the level of *p* < 0.05 was considered statistically significant).

## 3. Results

### 3.1. General Characteristics

The 337 patients with a working diagnosis of MINOCA were included in this study. According to the main assumption, all patients were divided into three groups, with regard to the extent of myocardial injury expressed by an increase in hs-TnI. The first group consisted of 87 (25.8%) consecutive patients with a mild hs-TnI increase. In the second group, we included 77 (22.8%) consecutive patients with a moderate hs-TnI increase. The third group comprised 173 patients (51.3%) with a severe hs-TnI increase. Considering the three selected groups of patients included in the study, the extent of troponin increase was inversely correlated with age. Patients assigned to the first group were significantly older in comparison to the other two groups (69.1 ± 10.62 years vs. 64 ± 12.91 years vs. 62.6 ± 16.37 years; *p* = 0.008). There were no significant differences between groups in the rates obtained by females, although there was a remarkable over-representation of women. 

### 3.2. Clinical Characteristics

Patients from the first group had a greater burden of cardiovascular risk factors, which included hyperlipidemia (66.3% vs. 45.5% vs. 35.9%; *p* < 0.001), arterial hypertension (83.7% vs. 83.1% vs. 67.8%; *p* = 0.004), and diabetes (34.9% vs. 20.8% vs. 22.2%; *p* = 0.052) when compared to the remaining two groups with greater troponin increases. Pharmacotherapy and other clinical indices are presented in Table 1.

### 3.3. Biochemical Parameters

With regard to biochemical parameters, we noticed significantly greater white blood cells counts in the third group when compared to the remaining ones (8.18 ± 2.98·10^3^/µL vs. 8.8 ± 3.12·10^3^/µL vs. 9.98 ± 4.8·10^3^/µL; *p* = 0.02). Total serum cholesterol concentration was significantly greater among patients from the groups characterized with higher troponin increases (4.15 ± 1.02 mmol/L vs. 4.75 ± 1.1 mmol/L vs. 4.5 ± 1.1 mmol/L; *p* = 0.01); Table 1. 

### 3.4. Type of Acute Coronary Syndrome, Electrocardiography, and Echocardiography

The rate of patients with a STEMI working diagnosis was significantly greater in the third group compared to the other two groups (9.3% vs. 11.5% vs. 21.4%; *p* < 0.001). Significantly lower mean left ventricle ejection fraction (LVEF) was noticed in the first group in comparison to the second and third groups (43.6 ± 15.8% vs. 52.1 ± 11.8% vs. 47.8 ± 14.3%; *p* < 0.001; Table 2).

### 3.5. Coronary Artery Angiography

We did not observe significant differences in the evaluated coronary artery angiography indices except for the contrast slow-flow phenomenon, which was found significantly more often in the third group than in the remaining two (2.3% vs. 2% vs. 11%; *p* = 0.02; Table 2).

### 3.6. Working Etiology and Discharge Diagnosis

For a significant number of patients in each group, the underlying cause of illness remained unclear, and the parentage of patients with unknown etiology was greatest in the third group when compared to the remaining two (16.1% vs. 23.4% vs. 33.4%; *p* < 0.001). The percentage of patients with type 2 myocardial infarction was significantly greater in the first, when compared to the second and third group (43.7% vs. 29.8% vs. 10.4%, *p* < 0.001) Nonetheless, the rate of patients with Takotsubo cardiomyopathy as the potential etiology of myocardial injury at admission was found most often in the third group when compared to the remaining two (0% vs. 5.2% vs. 15.5%; *p* < 0.001; Table 3).

The percentage of patients with Takotsubo cardiomyopathy diagnosis at discharge from hospital remained most frequent in the third group in comparison to the others (12.1% vs. 1.3% vs. 0%; *p* < 0.001). The rate of patients with a final diagnosis of arterial hypertension (8.1% vs. 6.5% vs. 2.3%; *p* = 0.01) and heart failure (31% vs. 3.9% vs. 11.5%; *p* < 0.001) was greatest in the first group of patients compared to the other groups (Table 4).

### 3.7. Clinical Outcomes

**Figure 1:**
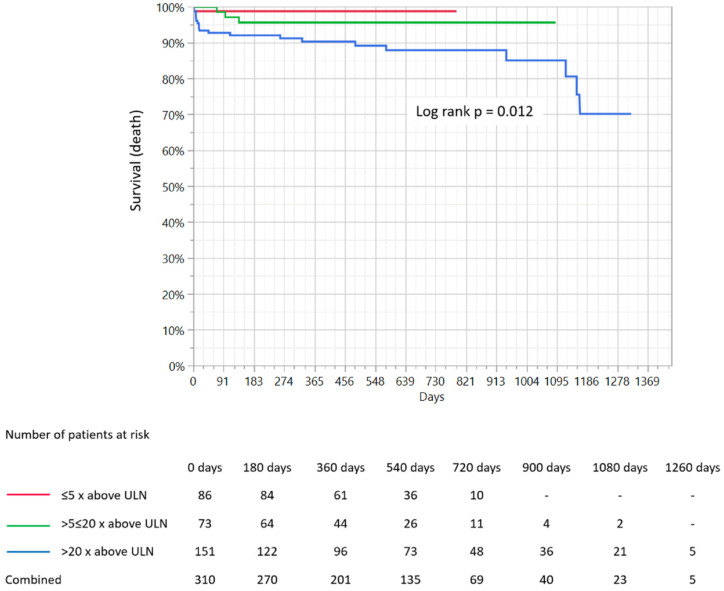
Comparison of Kaplan–Meier survival curves for three groups of patients depending on the extent of troponin I increase and presentation of mortality rates. ULN-upper limit of normal.

**Figure 2:**
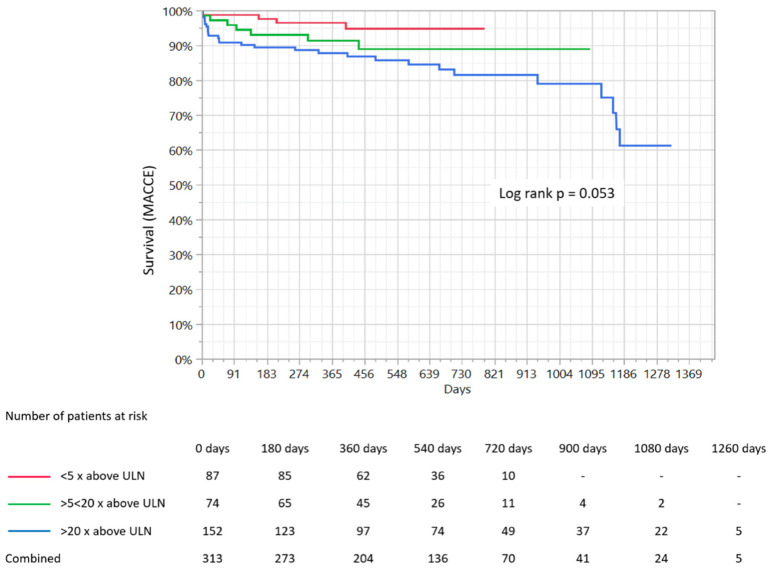
Comparison of Kaplan–Meier survival curves for three groups of patients depending on the extent of troponin I increase and presentation of MACCE rates. MACCE-major adverse cardiac and cerebrovascular events, ULN-upper limit of normal.

The mean duration of the follow-up period was 516.1 ± 299.9 days and did not differ significantly between groups (482 ± 173 days vs. 461 ± 245 days vs. 566 ± 382 days; *p* = 0.23). The follow-up was available for 94.5% of participants. The overall rate of MACCE was 12.1%, while the death rate was 7.5%, myocardial infarction rate was 9.5%, re-PCI 1.1% and cerebral stroke/TIA was 1.1%. The distribution of particular components of MACCE in the selected groups according to the increase in hs-TnI level is presented in Table 4. The overall incidence of MACCE was significantly greater in the third group compared to the other two (4.6% vs. 9.4% vs. 18.4%; *p* = 0.005). The overall all-cause mortality rate was also significantly greater in the third group in comparison to the first and second (1.1% vs. 4% vs. 13.1%; *p* = 0.001). Kaplan–Meier survival curves demonstrated that mortality rate during the follow-up period was significantly greater in the third group in comparison to the first and second (*p* = 0.01; Figure 1), while the MACCE rate was also greatest in the third group based on Kaplan–Meier curve analysis. However, its level did not reach statistical significance (*p* = 0.053; Figure 2).

### 3.8. Predictors of Clinical Outcomes

Univariate cox regression analysis demonstrated that the following were among predictors of increased risk of death rate during the follow-up period: age (*p* = 0.02); longer duration of hospitalization (*p* = 0.003); white blood cell (WBC) count (*p* = 0.02); serum creatinine concentration (*p* = 0.01); chronic kidney failure defined as estimated glomerular filtration rate (eGFR) <60 mL/min/1.72 m^2^ (*p* = 0.003); cardiac arrest before admission (*p* < 0.001); chronic obstructive pulmonary disease (COPD)/bronchial asthma (*p* = 0.03); therapy with corticosteroids (*p* = 0.001); hormone replacement therapy (*p* = 0.007); alcohol abuse (*p* < 0.001); ST segment elevation/left bundle branch block when compared to other ischemic electrocardiographic changes or no changes (*p* = 0.01); presence of regional akinesis in echocardiography compared to other contractility disorders or lack thereof (*p* = 0.04); LVEF lower than 40% (*p* = 0.01); tachyarrhythmias/atrioventricular conduction disorders (*p* = 0.03); pericardial effusion (*p* < 0.001); and serum hs-TnI elevation more than 20-times above the upper normal limit compared to lower elevation greater than 5-times above the upper normal limit (*p* = 0.02). Among predictors of lower all-cause mortality rate, the following were found: chest pain at admission (*p* < 0.001); arterial hypertension (*p* = 0.01); hyperlipidemia (*p* = 0.01); therapy with beta-blockers (*p* = 0.01); body mass (*p* = 0.008); body mass index (*p* = 0.02); greater LVEF (*p* = 0.02); blood hemoglobin concentration (*p* = 0.002); higher platelet count (*p* = 0.02); and D-dimer concentration (*p* = 0.01).

Using univariate cox regression analysis, the following variables were identified as predictors of increased MACCE rate during the follow-up period: cardiac arrest before admission (*p* < 0.001); kidney failure assessed as eGFR < 60 mL/min. (*p* = 0.02); treatment with corticosteroids (*p* = 0.02); hormone replacement therapy (*p* = 0.02); alcohol abuse (*p* = 0.01); pericardial effusion (*p* = 0.002); serum hs-TnI level greater than 20-times above the upper norm in comparison to an hs-TnI level lower than 5-times above the upper normal limit (*p* = 0.02); duration of hospitalization (*p* = 0.006); serum creatinine concentration (*p* < 0.001); maximal level of creatinine kinase myocardial band (*p* = 0.003); and WBC count (*p* = 0.04). Predictors of lower MACCE rate were: presence of chest pain at admission (*p* < 0.001); hyperlipidemia (*p* = 0.04); body mass (*p* = 0.01); body mass index (*p* = 0.04); blood hemoglobin concentration (*p* = 0.01); and level of low-density lipoproteins (*p* = 0.02). 

**Figure 3:**
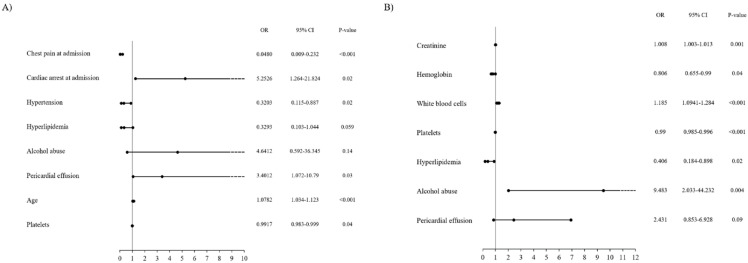
(**A**) Multivariable cox regression analysis regarding potential predictors of the occurrence of deaths during the follow-up period. (**B**) Multivariable cox regression analysis regarding potential predictors of the occurrence of MACCEs during the follow-up period. CI-confidence interval, OR-odds ratio.

Multivariable analysis allowed to identify the following variables as significant independent predictors of increased death rate during the follow-up period: cardiac arrest at admission (*p* < 0.001); pericardial effusion (*p* = 0.03); and age (*p* = 0.04). Among the significant predictors of lower all-cause mortality rate, we found: chest pain at admission (*p* < 0.001); arterial hypertension (*p* = 0.02); and greater platelet count (*p* = 0.04). This information is presented in Figure 3A. The model was characterized by very high goodness-of-fit, with a C-statistic of 0.91 (bootstrap value of 0.88). Proportional hazard assumptions were met (*p* = 0.31).

Multivariable analysis confirmed the following to be among significant independent predictors of increased MACCE risk during the follow-up period: greater serum creatinine concentration (*p* = 0.001); greater WBC count (*p* < 0.001); and alcohol abuse (*p* = 0.004). Among the significant predictors of lower MACCE occurrence during the follow-up period the following were confirmed: higher hemoglobin concentration (*p* = 0.04) and presence of hyperlipidemia (*p* = 0.02). This is presented in Figure 3B. The model was characterized by high goodness-of-fit with a C-statistic of 0.73 (bootstrap value of 0.69). Proportional hazard assumptions were met (*p* = 0.13).

## 4. Discussion

The main finding of the presented study, obtained on the basis Kaplan–Meier survival curve analysis, is that patients in the group with the greatest hs-TnI increase are related to significantly higher all-cause mortality rates during the follow-up period. A similar relationship was noted for the overall MACCE rate, however, it was not found to be statistically significant. Furthermore, this relationship was no longer significant for death and MACCE rates in multivariable analysis. Among the factors significantly related to all-cause mortality estimated in multivariable analysis during the follow-up period, we found cardiac arrest and chest pain at admission, pericardial effusion, age, arterial hypertension, and blood platelet count. Moreover, serum creatinine concentration, WBC count, alcohol abuse, hemoglobin concentration, and hyperlipidemia were found to be among the factors significantly related to MACCE rate during the follow-up. This was further confirmed in multivariate analysis.

As demonstrated in previously published studies, higher hs-TnI levels are associated with an underlying burden of coronary atherosclerosis, more rapid progression of coronary atherosclerosis, as well as higher risk of all-cause mortality and the incidence of cardiovascular events in patients undergoing cardiac catheterization and without evidence of ACS [11]. It has been observed that even a slight increase in troponin values above the upper normal limit is related to increased mortality rate during the follow-up period [12]. The extent of hs-TnI increase during hospitalization reflects the size of myocardial injury. It is simultaneously correlated with long-term prognosis, which is poorer in patients with a greater TnI increase during the first 24 h among patients with MI [13]. The duration of elevated TnI values persisting after MI is associated with increased follow-up mortality rate [1]. The role of TnI level prognostic value in patients hospitalized due to ACS with non-obstructive coronary artery disease has also been investigated [6]. Hs-TnT levels in MINOCA patients were found to be strong predictors of all-cause and cardiovascular mortality, as well as major adverse cardiac events (MACE) [6]. Hs-TnT value was demonstrated as a predictor of readmissions for heart failure but not non-fatal myocardial infarction or stroke [6]. In that study, patients with previously known coronary atherosclerosis or PCIs were excluded from the MINOCA group. The relationship between the extent of troponin increase and 1-year mortality in the MINOCA and MI-CAD groups suggested that hs-TnT was at least as prognostic in patients with MINOCA as in MI-CAD [6]. Despite the fact that the MINOCA group of patients differs in many aspects related to etiology compared to the MI-CAD group, in addition to the relationship between the degree of increase in peri-infarction TnI concentration and long-term clinical outcomes, such a relationship has been demonstrated for a number of other factors. These may include age or diabetes, which are typical follow-up risk factors among patients with MI-CAD [7]. Although a significant relationship between the degree of TnI increase and mortality was demonstrated in Kaplan–Meier curve analysis, in the current study, multivariable analysis did not confirm the significance of such a relationship, showing other factors as more important depending on the endpoint. Publications regarding long-term outcomes in patients with MINOCA predictors are less common than for patients with MI-CAD. Nevertheless, in another study, in which more than 9000 MINOCA patients were observed over a 4.5-year follow-up period, the mortality rate was 14%, while MACE was 24% [7]. We have confirmed older age, diabetes, hypertension, current smoking, previous MI, previous stroke, peripheral vascular disease, COPD, reduced LVEF, lower level of total cholesterol, and higher level of creatinine to be among the independent predictors of MACE [7]. In the case of independent predictors for all-cause deaths, age, current smoking, diabetes, cancer, COPD, previous stroke, reduced LVEF, lower level of total cholesterol, and higher levels of creatinine and c-reactive protein were found [7].

The negative impact of anemia on long-term mortality among 2011 patients with MI-CAD treated with PCIs was demonstrated in the study published by Colombo et al. [14]. Similar results were presented by Wańha et al. in a study including 1916 consecutive patients with coronary artery disease treated with PCI and stent implantation [15]. In comparison, in the current study, anemia was found to be a significant predictor of MACCE, but not death.

Furthermore, in this study, thrombocytopenia is another significant predictor of MACCE also mentioned in other studies concerning patients with MI-CAD [16]. However, in some studies, it has been shown that among patients with STEMI, an increased platelet count also worsens long-term prognosis within the context of a higher incidence of target lesion revascularizations (thrombotic complications) and the overall rate of MACE [17]. These results may indicate that, in patients with MI, the relationship between platelet counts and distant results may be U-shaped for patients with MI-CAD, whereas in the case of MINOCA, the frame considering the negative impact of increased platelet counts on distant results may have less significant impact [18].

Systemic inflammatory response is observed in ACS. A relationship between proinflammatory markers and clinical outcomes was found in patients with STEMI [19]. It has been proven that higher blood leukocyte levels are associated with higher mortality rate and in-hospital complications [20]. The higher WBC count notably correlated with intra-hospital deaths as well as long-term mortality [20]. Additionally, neutrophilia at admission was related to the significantly greater rate of adverse cardiac events in 228 patients with ACS during the mean follow-up period of 52 months [21].

In this study, higher cholesterol level appears to have had a protective effect in the MINOCA group of patients and is associated with fewer adverse events. Hypercholesterolemia is an established cardiovascular risk factor of CAD and future MACCE occurrence. Despite this fact, in the literature, we can find the so-called “hypercholesterolemia paradox”, in which blood serum cholesterol levels positively correlate with the beneficial influence of remnant-like lipoprotein particles [22]. This can be partially explained by their large share in the MINOCA group and the subgroup with more severe myocardial damage expressed as greater concentration of hs-TnI in patients with moderately and severely decreased LVEF. The presence of the hypercholesterolemia paradox in the group of patients with heart failure is already sanctioned [23,24].

There are no specific publications on pericardial effusion as a predictor of MACCE. In one published study, conducted among a group of 1732 patients with STEMI, it was observed that the presence of pericardial effusion in the period following primary PCI was not independently associated with mortality [25]. To the contrary, in another study, it was demonstrated that moderate-to-large pericardial effusion complicating STEMI was a common finding (almost 25%) and is related to more severe infarcts with subsequently significantly increased MACE rates during the 1-year follow-up period. As a consequence, moderate-to-large pericardial effusion was found to be a marker of poor outcomes in patients with STEMI [26]. In the current study, pericardial effusion was an independent predictor of increased all-cause mortality during the follow-up. This could be explained, at least in part, by the presence of patients with myocarditis and concomitant pericarditis, and their influence on clinical outcomes.

Although there are reports on the positive effects of moderate alcohol consumption due to the so-called U-shape relationship of its consumption with MACCE, alcohol abuse seems to have a negative impact on both MINOCA and MI-CAD patients in terms of higher MACCE rates during the follow-up period [27]. In patients with a MINOCA diagnosis, this is mainly attributed to the negative effect of alcohol consumption on the higher rate of heart failure and related admissions to hospital due to exacerbations [28].

Renal failure expressed as elevated markers of kidney function impairment in the serum is a well-known factor related to poorer clinical outcomes in patients with MI-CAD and treated with primary PCI. Furthermore, this relationship was also confirmed in the current study on MACCE rate [29].

In patients with AMI, age seems to have greater negative impact on long-term clinical outcomes in older patients, and this was confirmed to be a predictor of increased mortality during the follow-up period in the present analysis [30].

The group of patients with MINOCA is a specific cohort, and one of the typical features of this cohort of patients is greater percentage of females in comparison to male patients with MI-CAD. It has been demonstrated that women with chest pain and suspected coronary atherosclerosis less often present confirmed coronary atherosclerosis compared to males [31]. Johnson et al. demonstrated that females presenting non-obstructive coronary arteriosclerosis and recurrent chest pain during post-angiography 1-year follow-up, were at a higher risk of cardiovascular events [32]. Patients with recurrent chest pain and no relevant obstructive coronary atherosclerosis were found to more frequently present impaired coronary flow reserve flow and more advanced atherosclerosis with positive remodeling in intra-vascular ultrasound [33,34]. However, the lower all-cause mortality rate in patients presenting chest pain at admission in the group of patients with MINOCA is probably mostly related to delayed diagnosis and less advanced impairment of myocardial injury in terms of LVEF and microvascular circulation.

## 5. Conclusions

While the relationship between clinical outcomes and the extent of hs-TnI increase during hospitalization in patients with a working diagnosis of MINOCA remains visible, it is not as strong as it is in patients with obstructive coronary atherosclerosis. Prognosis in the group of patients with a working diagnosis of MINOCA at baseline is different and depends on many factors, which is mainly determined by the very diverse etiology and pathomechanisms responsible for ACS in this group of patients. Therefore, in the case of prognosis assessment in this group of patients and the decision to introduce the most appropriate treatment in order to improve its results, multi-faceted diagnostics is used to determine the dominant etiology and pathomechanism of myocardial infarction.

The use of the troponin conversion factor relative to their upper limit of the norm, in exchange for their continuous values, could have introduced bias, but in our opinion, unification of the results. Taking into account different methods of determining troponins depending on the centers and time of determination, was a priority. The use of the working MINOCA diagnosis to calculate all-cause mortality and MACCE predictors may introduce some distortions or inaccuracies, because one of these patients will eventually be removed from the MINOCA group to other groups, e.g., myocarditis or takotsubo cardiomyopathy. However, in order to carry out diagnostics in the MINOCA group, it would have to be limited to patients who have been diagnosed with other potential causes of myocardial injury. Such adiagnosis often takes several months and includes a number of tests, including those for hypercoagulability. A great limitation regarding interpretation of the research results is also associated with the relatively small group of patients, but on the other hand, in this manner, it is possible to achieve conclusions on local trends and tendencies which may contribute to determining the appropriate treatment and care over this group of patients, while improving long-term treatment results. In the present publication, we do not present the exact results of the physiological studies on coronary circulation (fractional flow reserve), intravascular ultrasound, or the exact division depending on the thickness of the atherosclerotic lesion walls and the degree of their dissemination within the coronary arteries. This may be significantly related to the assessed study endpoints, introducing significant bias.

## Figures and Tables

**Table 1:** General patient characteristics and biochemical analysis according to the increase in high-sensitivity cardiac I troponin.

Clinical Characteristics	Concentration of High-Sensitivity Cardiac I Troponin (Increase—Times above the Upper Normal Limit)			*p*-Value
	≤5	>5 to ≤20	>20	
**Age, years**	69.1 ± 10.6	63.9 ± 12.9	62.6 ± 16.4	0.008
Hospitalization duration, days	4.58 ± 2.38	5.38 ± 3.45	6.06 ± 3.42	0.02
Gender, female	41 (47.1)	39 (50.6)	79 (45.7)	0.76
Arterial hypertension	72 (83.7)	64 (83,1)	116 (67.8)	0.004
Hyperlipidemia	57 (66.3)	35 (45.4)	61 (35.9)	<0.001
Diabetes	30 (34.9)	16 (20.8)	38 (22.2)	0.052
Kidney failure	15 (17.6)	7 (9.1)	19 (11.2)	0.2
Atrial fibrillation	17 (19.5)	15 (19.5)	25 (14.5)	0.46
COPD/Asthma	9 (10.5)	8 (10.4)	14 (8.2)	0.77
Autoimmune disease	2 (2,3)	5 (6.5)	7 (4.1)	0.45
Oncological disease	8 (9.3)	7 (9.1)	11 (6.5)	0.65
Hypercoagulable state	6 (7)	3 (3.9)	3 (1.8)	0.13
Smoking	22 (26.5)	19 (35.2)	12 (29.3)	0.55
Alcohol abuse	2 (2.3)	2 (2.6)	7 (4.3)	0.67
Prior myocardial infarction	30 (34.5)	16 (20.8)	37 (21.5)	0.04
Prior PCI	32 (36.8)	12 (15.6)	27 (15.8)	<0.001
Cardiac arrest	3 (3.4)	0 (0)	15 (8.8)	0.01
Prior cerebral stroke/TIA	6 (6.9)	4 (5.2)	12 (7)	0.86
Prior DVT/PE	2 (2.3)	0 (0)	4 (2.3)	0.4
Pharmacotherapy				
Acetyl salicylic acid	33 (55)	26 (42.6)	33 (22.9)	0.04
P_2_Y_12_ blockers	14 (25)	8 (13.6)	10 (7)	0.002
Anticoagulants	17 (28.3)	15 (25)	14 (9.9)	0.001
Beta-blocker	38 (59.4)	28 (43.7)	42 (29.2)	<0.001
Statins	38 (59.4)	27 (44.3)	36 (25)	<0.001
Biochemical Analyses				
White blood cells, 10^3^/µL	8.18 ± 2.98	8.8 ± 3.12	9.98 ± 4.8	0.02
Platelet count,·10^3^/µL	225.9 ± 98	239.8 ± 79.8	220.5 ± 79.8	0.12
Hemoglobin, g/dL	13.5 ± 1.6	13.1 ± 1.8	12.9 ± 2.1	0.15
C-reactive protein, mg/L	13.3 ± 9.8	44.4 ± 53.9	41.1 ± 59.1	0.16
Total Cholesterol, mmol/L	4.15 ± 1.02	4.75 ± 1.1	4.5 ± 1.1	0.01
Cholesterol HDL, mmol/L	1.25 ± 0.4	1.34 ± 0.4	1.24 ± 0.4	0.45
Cholesterol LDL, mmol/L	2.24 ± 0.8	2.66 ± 1	2.58 ± 1.1	0.4
Triglycerides, mmol/L	1.48 ± 0.8	1.68 ± 1.1	1.49 ± 0.8	0.79
eGFR < 60 mL/min.	20 (25.6)	13 (19.1)	34 (25.9)	0.53
Creatinine, µmol/L	76.5 ± 34.6	68.9 ± 14.5	83.56 ± 43.9	0.23
CK-MB at admission, U/I	1.5 ± 3.1	11.7 ± 15.2	44 ± 75	<0.001
CK-MB max., U/I	30.4 ± 23.1	28.1 ± 16.4	68.6 ± 160	<0.001

CK-MB—creatine kinase myocardial band, COPD—chronic obstructive pulmonary disease, DVT—deep venous thrombosis, eGFR—estimated glomerular filtration rate, HDL—high-density lipoproteins, LDL—low-density lipoproteins, PCI—percutaneous coronary intervention, PE—pulmonary embolism, TIA—transient ischemic attack.

**Table 2:** Clinical presentation, electrocardiographic, echocardiographic, and coronary angiographic data.

	Concentration of High-Sensitivity Cardiac I Troponin (Increase—Times above the Upper Normal Limit)			*p*-Value
	≤5	>5 to ≤20	>20	
Type of Myocardial Infarction at Admission—Working Diagnosis				
STEMI	8 (9.3)	9 (11.5)	37 (21.4)	<0.001
NSTEMI	69 (80.2)	68 (87.2)	136 (78.6)	
Unstable angina	9 (10.5)	0	0	
Electrocardiography				
ST segment elevation/LBBB	13 (15.3)	18 (23.4)	44 (25.6)	0.17
ST segment depression	21 (24.7)	20 (26)	29 (16.9)	0.16
T-wave inversion	15 (20.3)	14 (18.7)	34 (20.1)	0.95
Echocardiography				
LVEF ≥ 40%	49 (59.8)	60 (80)	114 (66,67)	0.02
Mean LVEF	43.6 ±15.8	52.1 ±11.8	47.8 ±14.3	0.001
Normal contractility	33 (39.8)	30 (40)	41 (24.4)	0.01
Present hypokinesis	32 (38.5)	26 (35.1)	72 (42.9)	0.05
Present akinesis	18 (21.7)	18 (24.3)	55 (32.7)	0.13
Pericardial effusion	2 (2.4)	5 (6.8)	15 (8.9)	0.15
Coronary Angiography				
Vascular access, radial	65 (75.6)	54 (76.1)	115 (78.8)	0.82
Non-stenotic plaques	65 (74.6)	59 (76.6)	119 (75.8)	0.95
Slow-flow contrasts	2 (2.3)	4 (5.2)	19 (11)	0.02
Eccentric plaque	2 (2.3)	3 (3.9)	1 (0.6)	0.17
Myocardial bridges	4 (4.6)	4 (5.2)	10 (5.8)	0.92
Arterial spasm	1 (1.1)	0 (0)	5 (2.9)	0.42
Thrombus	0 (0)	0 (0)	2 (1.1)	0.38

LBBB-left bundle branch block, LVEF—left ventricle ejection fraction, NSTEMI—non-ST-segment elevation myocardial infarction, STEMI—ST-segment elevation myocardial infarction.

**Table 3:** Working etiology of myocardial infarction determined during hospitalization according to increase in high-sensitivity cardiac I troponin.

Etiology	Concentration of High-Sensitivity Cardiac I Troponin (Increase—Times above the Upper Normal Limit)			*p*-Value
	≤5	>5 to ≤20	>20	
Unknown	14 (16.1)	18 (23.4)	58 (33.4)	0.008
Arterial spasm	0 (0)	1 (1.3)	4 (2.3)	0.34
Myocarditis	1 (1.2)	5 (6.5)	14 (8.1)	0.07
HCM	2 (2.3)	1 (1.3)	2 (1.2)	0.76
Takotsubo cardiomyopathy	0 (0)	4 (5.2)	27 (15.5)	<0.001
Slow-flow phenomenon	0 (0)	2 (2.6)	8 (4.6)	0.11
AV conduction disorders	3 (3.4)	3 (3.9)	2 (1.2)	0.31
Aortic dissection	0 (0)	1 (1.3)	1 (0.6)	0.55
Tachyarrhythmias	10 (12.6)	6 (7.8)	19 (11)	0.69
Atrial fibrillation	10 (12.6)	6 (7.8)	15 (8.7)	0.67
Arterial hypertension	13 (14.9)	9 (11.7)	4 (2.3)	<0.001
Anemia	0 (0)	0 (0)	2 (1.2)	0.38
Oncological embolization	1 (1.2)	1 (1.3)	3 (1.7)	0.92
Myocardial bridge	2 (2.3)	2 (2.6)	3 (1.7)	0.89
PE/DVT	0 (0)	0 (0)	1 (0.6)	0.62
Muscular dystrophy	0 (0)	0 (0)	1 (0.6)	0.62
Cerebral stroke	0 (0)	0 (0)	1 (0.6)	0.62
Alcoholic cardiomyopathy	0 (0)	0 (0)	1 (0.6)	0.62
Aortic valve stenosis	2 (2.3)	1 (1.3)	2 (1.2)	0.76
Antiphospholipid syndrome	0 (0)	0 (0)	1 (0.6)	0.62
Vasculitis	0 (0)	0 (0)	1 (0.6)	0.62

AV—atrio-ventricular, DVT—deep venous thrombosis, HCM—hypertrophic cardiomyopathy, PE—pulmonary embolism.

**Table 4:** Diagnosis at discharge and follow-up outcomes.

Selected Indices	Concentration of High-Sensitivity Cardiac I Troponin (Increase—Times above the Upper Normal Limit)			*p*-Value
	≤5	>5 to ≤20	>20	
Diagnosis at Discharge from Hospital				
Myocarditis	1 (1.1)	4 (5.2)	13 (7.5)	0.09
Takotsubo cardiomyopathy	0 (0)	1 (1.3)	21 (12.1)	<0.001
Arrhythmias	5 (5.8)	5 (6.5)	4 (2.3)	0.21
Atrial fibrillation	5 (5.8)	5 (6.5)	3 (1.7)	0.11
Arterial hypertension	7 (8.1)	5 (6.5)	2 (1.2)	0.01
Venous thromboembolic disease	0 (0)	0 (0)	1 (0.6)	0.62
NSTEMI	38 (43.7)	47 (61)	83 (48)	0.06
STEMI	3 (3.4)	4 (5.2)	14 (8.1)	0.31
Heart failure	27 (31)	3 (3.9)	20 (11.5)	<0.001
Type 2 myocardial infarction	5 (5.8)	6 (7.8)	12 (6.9)	0.87
Hypertrophic cardiomyopathy	1 (1.1)	1 (1.3)	1 (0.6)	0.81
Myocardial bridge	0 (0)	1 (1.3)	1 (0.6)	0.55
Cerebral stroke	0 (0)	0 (0)	1 (0.6)	0.62
Follow-Up				
Mean time of follow-up, days	482 ± 173	461 ± 245	566 ± 382	0.23
MACCE	4 (4.6)	7 (9.4)	28 (18.4)	0.005
Overall all-cause mortality	1 (1.1)	3 (4)	20 (13.1)	0.001
In-hospital all-cause mortality	1 (1.1)	1 (1.3)	6 (3.5)	0.39
Myocardial infarction	4 (4.6)	3 (4)	24 (15.8)	0.003
PCI	0 (0)	0 (0)	4 (2.6)	0.11
Cerebral stroke/TIA	0 (0)	3 (4)	0 (0)	0.007

MACCE—main adverse cardiac and cerebrovascular events, PCI—percutaneous coronary intervention, TIA—transient ischemic attack
